# Styrene maleic acid recovers proteins from mammalian cells and tissues while avoiding significant cell death

**DOI:** 10.1038/s41598-019-51896-1

**Published:** 2019-11-25

**Authors:** Andrew J. Smith, Kathleen E. Wright, Stephen P. Muench, Sophie Schumann, Adrian Whitehouse, Karen E. Porter, John Colyer

**Affiliations:** 1 School of Biomedical Sciences, Faculty of Biological Sciences, University of Leeds, Woodhouse Lane, Leeds, LS2 9JT United Kingdom; 2 Centre for Human and Applied Physiological Sciences and Centre for Stem Cell and Regenerative Medicine, Guy’s Campus, King’s College London, London, SE1 1UL United Kingdom; 3 The Astbury Centre for Structural Molecular Biology, Faculty of Biological Sciences, University of Leeds, Woodhouse Lane, Leeds, LS2 9JT United Kingdom; 4 School of Molecular and Cellular Biology, Faculty of Biological Sciences, University of Leeds, Woodhouse Lane, Leeds, LS2 9JT United Kingdom; 5 Division of Cardiovascular & Diabetes Research, Leeds Institute of Cardiovascular & Metabolic Medicine (LICAMM), Faculty of Medicine and Health, University of Leeds, Woodhouse Lane, Leeds, LS2 9JT United Kingdom

**Keywords:** Membrane proteins, Proteomic analysis, Diagnostic markers

## Abstract

Detection of protein biomarkers is an important tool for medical diagnostics, typically exploiting concentration of particular biomarkers or biomarker release from tissues. We sought to establish whether proteins not normally released by living cells can be extracted without harming cells, with a view to extending this into biomarker harvest for medical diagnosis and other applications. Styrene maleic acid (SMA) is a polymer that extracts nanodiscs of biological membranes (containing membrane proteins) from cells. Hitherto it has been used to harvest SMA-lipid-membrane protein particles (SMALP) for biochemical study, by destroying the living cellular specimen. In this study, we applied SMA at low concentration to human primary cardiovascular cells and rat vascular tissue, to ‘biopsy’ cell proteins while avoiding significant reductions in cell viability. SMA at 6.25 parts per million harvested proteins from cells and tissues without causing significant release of cytosolic dye (calcein) or reduction in cell viability at 24 and 72 hours post-SMA (MTT assay). A wide range of proteins were recovered (20–200 kDa) and a number identified by mass spectrometry: this confirmed protein recovery from plasma membrane, intracellular membranes and cell cytosol without associated cell death. These data demonstrate the feasibility of non-lethally sampling proteins from cells, greatly extending our sampling capability, which could yield new physiological and/or pathological biomarkers.

## Introduction

Current cell-based biopsy measurements rely on using destructive techniques to remove and isolate particular cellular components, especially those housed within a membrane environment. While these techniques can provide a useful approach for obtaining valuable information, with a view to obtaining specific biomarkers, a non-destructive means of actively isolating biomarkers would open avenues for new marker isolation techniques, thereby advancing disease diagnoses to earlier stages, or for safely obtaining information from small, irreplaceable cell samples.

We have examined the use of styrene maleic acid (SMA), an amphipathic polymer, in a previously unexplored context: to determine if it could obtain cell proteins while retaining cell viability, in effect to non-lethally ‘biopsy’ cells. A key motivation for this study was to identify if such a method could build on the rapid growth of research into exosomes^1^ and microvesicles^2^, specifically that driven by the recognition of their potential as diagnostic markers^3,4^. The detection of disease biomarkers, in terms of the disease phase detected by these markers, has advanced from the reactive detection of markers of cell destruction such as elevated cardiac troponin post-MI, to the prospective identification of disease markers that are released from cells in advance of symptoms^3,4^. The next level of advancing biomarker detection to an earlier stage of pathology would be to move from awaiting the passive shedding of such markers from cells to a process of actively collecting them. Achieving this will require means to obtain such proteins, while also capturing them in a stable configuration to permit subsequent analysis.

Isolating membrane proteins has a number of significant challenges, not least removing them from the membrane environment and then maintaining them in a stable state once removed. Traditionally this has been achieved through a detergent-based approach with numerous types of detergents available with differing properties^5^. These allow for membrane proteins to be stabilised without the need for the lipid bilayer and subsequent biophysical characterisation by, for example X-ray crystallography, electron microscopy and NMR^6^. More recently, it has been demonstrated that SMA isolates membrane proteins from a membrane environment in stable disc-shaped nanoparticles^7^, allowing preservation of proteins or protein complexes in a more native state, with a ‘cuff’ of lipid bilayer around the protein. Moreover, SMA-extracted proteins, as with those stabilised in more traditional detergents can be studied through a range of biophysical techniques^8–10^. This is applicable not only to bacterial membranes but has also been demonstrated to be successful for HEK cell membranes^11^.

Here we have investigated if an SMA approach could be applied to a range of cells for the extraction of membrane proteins for biochemical studies, in a non-lethal process in contrast to the destructive nature of current techniques^12^. While previous explorations of SMA function have provided a novel and valuable tool in the biochemical and structural biology context, this is to our knowledge the first demonstration of the ability of SMA to harvest proteins from cells non-lethally, most notably from human cells. Our first objective was to identify if such proteins could be retrieved from cells via SMA; if this proved successful, our second objective was to carry out a broad assessment of which proteins were sampled from cells and from which cellular locations, thereby obtaining a broad overview of the method’s potential for further utilisation. We elected to achieve these aims using human cells, to most rapidly assess validity for potential translational development. SMA was also able to recover proteins from intact tissue, in this case rat arterial blood vessel *ex vivo*, without causing apparent harm to the cells of the vessel.

## Results

### Application of SMA without significant cell death

**Figure 1:**
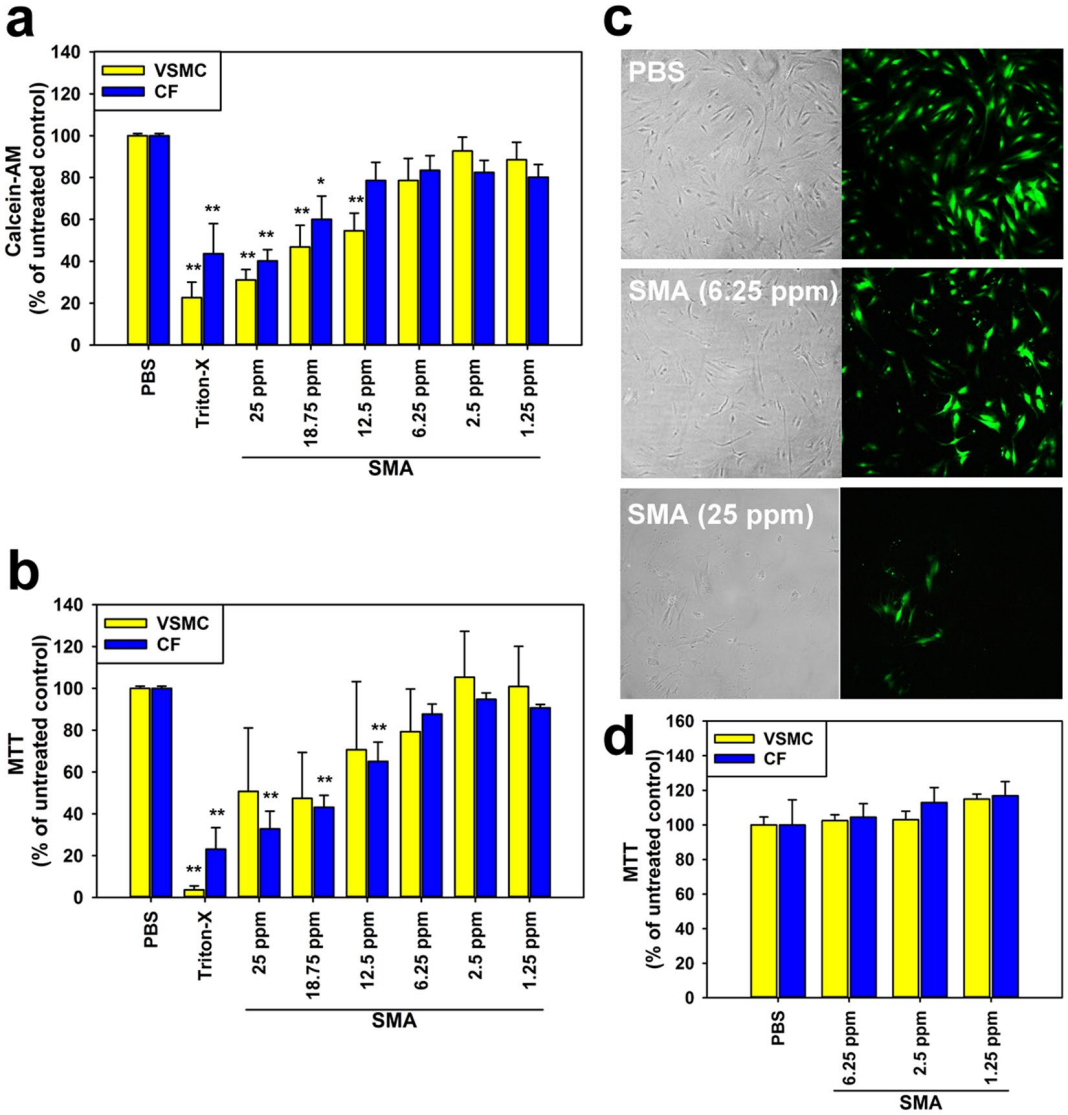
Low-dose styrene maleic acid can be applied to cells without significantly reducing viability. (**a**) Styrene maleic acid applied to human CFs or VSMCs for 10 minutes at 37 °C identified a range of concentrations that did not significantly reduce viability, with membrane integrity demonstrated by calcein-AM assay (ANOVA plus Tukey’s test *p < 0.05, **p < 0.01, n = 8). (**b**) Cell viability was confirmed 24 hours post-SMA application by MTT assay (ANOVA plus Tukey’s test **p < 0.01, n = 8). (**c**) Assay findings were consistent with morphological appearances on microscopy, with intact viable cells stained green with calcein-AM. (**d**) Cell viability for low-dose SMA concentrations was re-confirmed 72 hours post-SMA application by MTT assay (ANOVA plus Tukey’s test, n = 6).

The first objective was to determine whether there was an effective concentration range at which SMA could be applied to human cells without a significant impact on viability. To avoid misrepresentative results due to a particularly low vulnerability of one cell type, we examined two different types of primary cultured human cardiovascular cells in parallel: cardiac fibroblasts (CFs) and vascular smooth muscle cells (VSMCs), which were isolated according to our established methods^13,14^. A dual-assay approach was utilised to determine the impact of SMA on cell survival: cells were loaded with 10 µM calcein-AM prior to SMA application to determine impact on membrane integrity acutely, then MTT assays to determine cell viability were performed 24 hours post-SMA application to identify any delayed cell death. With a view to potential future applications of SMA cell ‘biopsy’ in both *in vitro* and *in vivo* contexts, a brief (but sufficiently long to ensure consistency across repeats) application of SMA (10 minutes) was used. Using a range of SMA concentrations, from 1.25 to 25 parts per million (ppm), we demonstrated that SMA applied at or below 6.25 ppm did not significantly reduce cell membrane integrity, as determined by the retention of calcein in the cell cytoplasm, in either cell type (Fig. 1a). Subsequent analysis using MTT assay confirmed no significant impact on cell viability 24 hours after SMA application at these levels (≤6.25 ppm; Fig. 1b) but with clear evidence of cell death at higher concentrations of SMA. Macroscopic appearances of cell phenotypes in culture were consistent with these assay findings, with normal cell morphologies following exposure to 0 or 6.25 ppm SMA corresponding to positive calcein staining (Fig. 1c). To exclude delayed cell death due to SMA application at or below 6.25 ppm, MTT assay 72 hours after SMA application confirmed no reduced cell viability (Fig. 1d).

### Low SMA concentration harvests proteins from cells

**Figure 2:**
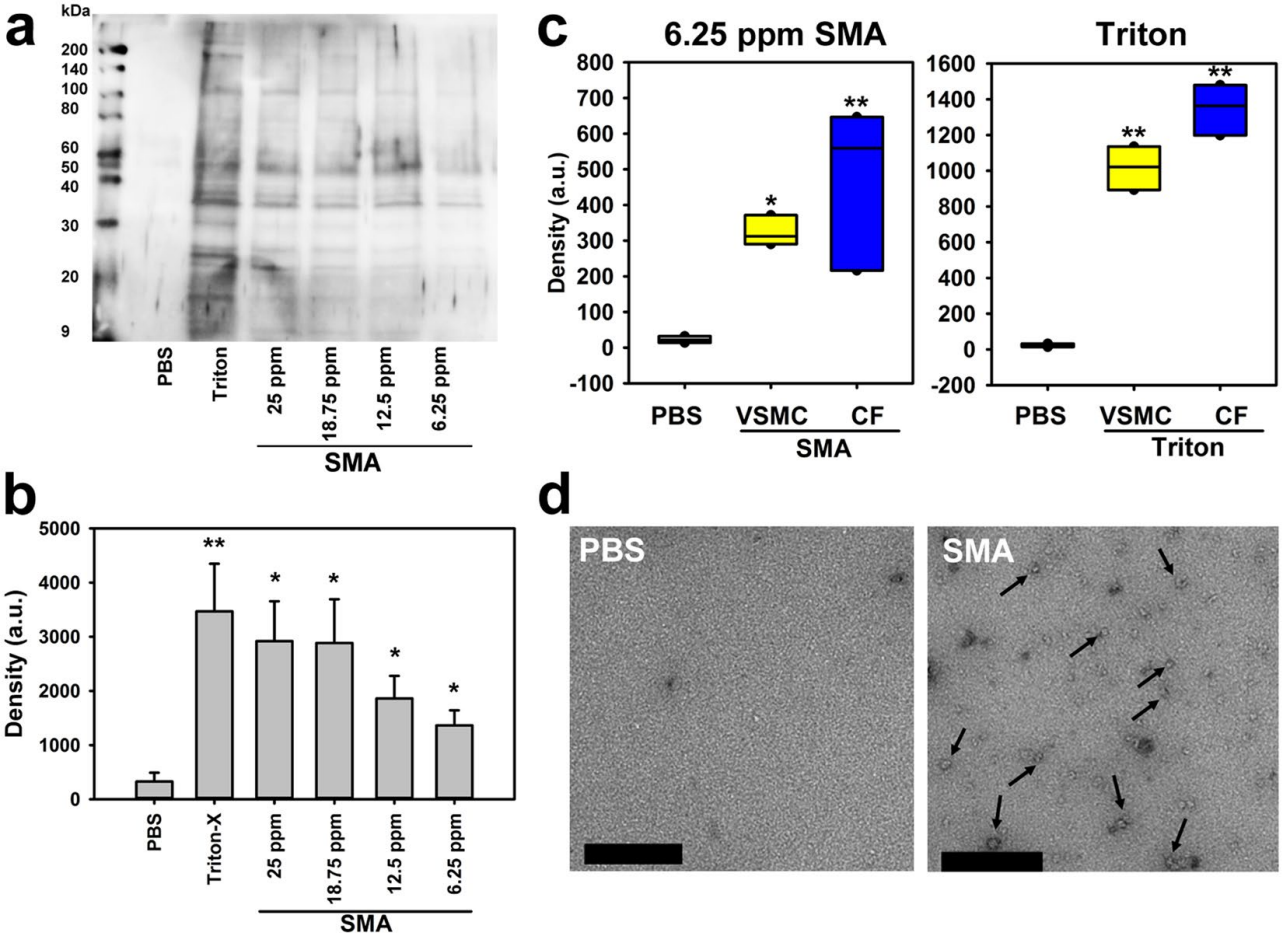
Low-dose styrene maleic acid application effectively samples proteins from cells. (**a**) Labelling of cells with biotin prior to 6.25 ppm SMA application allowed confirmation with biotin immunoblotting of the presence of a range of proteins (20–200 kDa) in the obtained suspension. (**b**) Proteins were absent from the final post-biotinylation Dulbecco’s PBS cell wash, as confirmed across repetitions (ANOVA plus Tukey’s test *p < 0.05, **p < 0.01 com*p*ared to Dulbecco’s PBS wash control, mean ± SEM, n = 3). (**c**) Gel analysis of SMA suspensions demonstrated requirement of SMA for protein collection in CFs and VSMCs, with no detectable protein seen in 0% SMA (Dulbecco’s PBS), with 6.25 ppm SMA obtaining quantifiable protein samples from both cell types and levels of protein obtained by 0.01% Triton-X for comparison (ANOVA plus Tukey’s test *p < 0.05, **p < 0.01, box and whiskers plots showing median ± 95% CI, n = 3). (**d**) The presence of SMALP particles in 6.25 ppm SMA suspension obtained from VSMCs was confirmed by electron microscopy, compared with their absence in 0% SMA (PBS, bar = 150 nm).

With a range of SMA concentrations thus identified as being sub-lethal to human cells, we then established whether this low-dose SMA application was also effective in sampling proteins from these cells. Firstly, VSMCs were biotinylated using a cell-impermeable biotin conjugation reagent before SMA application. SMA lipid particles (SMALP) obtained from cells by SMA application were collected in suspension, then analysed for biotinylated proteins using SDS-PAGE and immunoblotting. Biotinylated proteins over a range of sizes (approximately 20 to 200 kDa) were present in collected suspensions following application of SMA from 25 ppm to 6.25 ppm (Fig. 2a,b). As expected, biotinylated proteins were also harvested from cells treated with the detergent Triton-X. However, only residual background signal was seen in samples obtained using physiological buffer (Dulbecco’s PBS) with 0% SMA, showing that shedding of biotinylated proteins did not occur in the absence of SMA (Fig. 2a,b**)**.

Suspensions were collected following 6.25 ppm SMA application to non-biotinylated cells, with staining of SDS-PAGE gels identifying a similar (20 to 200 kDa) range of proteins. This protein sampling was seen from both cell types across repetitions, but not in the 0% SMA (negative control: PBS only) samples (Fig. 2c). Levels of proteins sampled in PBS containing 0.01% Triton-X were also assessed for comparative purposes (Fig. 2c). The presence of SMALPs (with a distinctive discoid morphology) and the identity of proteins harvested therein was determined by electron microscopy and mass spectrometry respectively. In the absence of exposure to SMA, no SMALP particles were observed by electron microscopy (Fig. 2d) and only a single or no protein was recovered from either CFs or VSMCs (Suppl. Fig. 1). In contrast, exposure of either cell type to 6.25 ppm SMA for 10 minutes resulted in the detection of SMALP nanodiscs using electron microscopy, with particle appearances consistent with SMALPs identified previously^11^ (Fig. 2d). Mass spectrometry identified in excess of 70 distinct proteins from each cell type, the characterisation of which was our next objective.

### Mapping of SMA sampling to cellular locations and cell type

**Figure 3:**
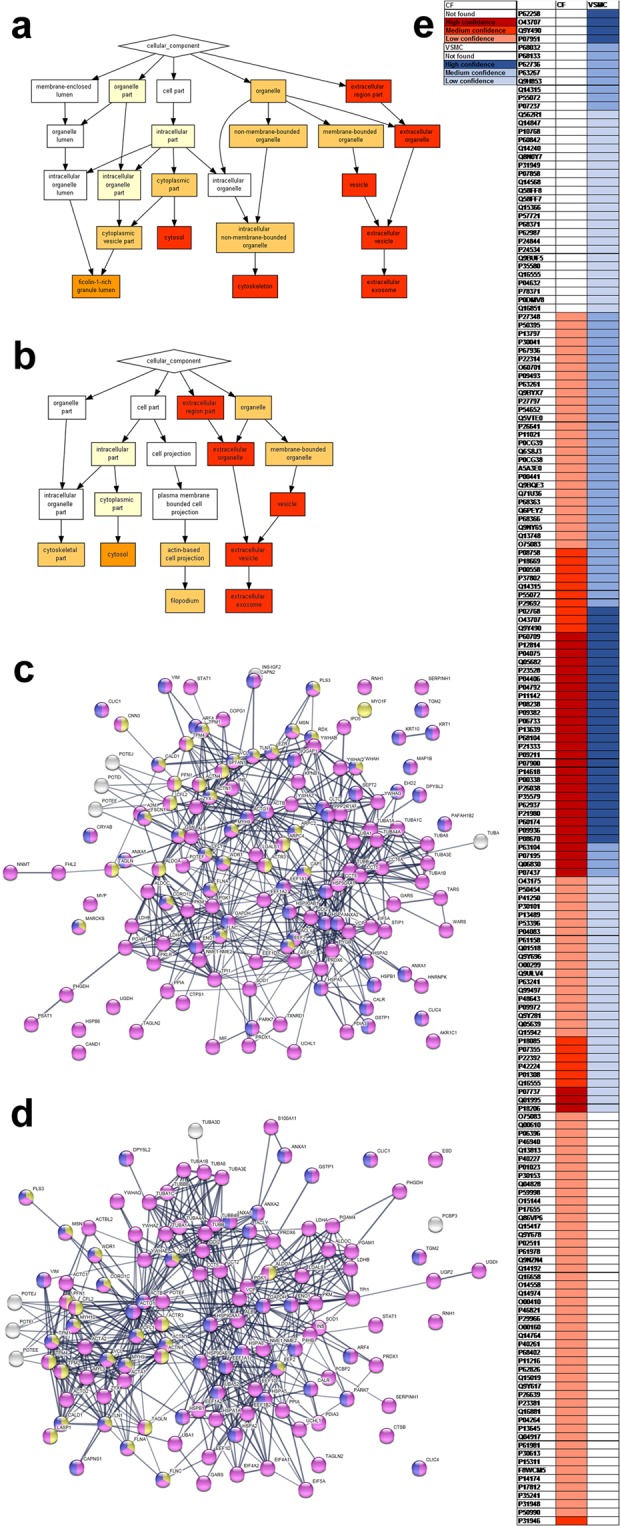
Mapping of cellular proteins and areas sampled by SMA biopsy. GO analysis of data identifying subcellular locations of all proteins sampled from (**a**) CFs (n = 3) and (**b**) VSMCs (n = 3), with consistent probabilities for locations of protein sampling in each cell population. (Colour code for GO maps, showing confidence of protein sampling from these locations: *p* > 10^−3^ white; *p* 10^−3^–10^−5^ beige; *p* 10^−5^–10^−7^ pale orange; *p* 10^−7^–10^−9^ dark orange; *p* < 10^−9^ red). (**c**) Mapping of known and predicted protein-protein interactions in SMA-biopsies from CFs by STRING analysis. Likely subcellular localisation indicated as follows: cytoplasm (purple, 93 proteins); plasma membrane (blue, 57 proteins); actin binding (yellow, 33 proteins). Line thickness indicates confidence of protein-protein interaction, a confidence threshold of 0.7 (high confidence) was used for all indicated interactions. (**d**) Mapping of known and predicted protein-protein interactions in SMA-biopsies from VSMCs by STRING analysis. Likely subcellular localisation indicated as follows: cytoplasm (purple, 115 proteins); plasma membrane (blue, 43 proteins); actin binding (yellow, 25 proteins). Line thickness indicates confidence of protein-protein interaction, a confidence threshold of 0.7 (high confidence) was used for all indicated interactions. (**e**) Heat map of proteins in SMA-biopsies specific to CFs (red) or VSMCs (blue) cells, and proteins sampled from both cell types (both colours).

With the primary objective of protein sampling from human cells without significant cell death achieved, we carried out analysis of the proteins recovered by SMA biopsy. Following application of SMA (6.25 ppm, 10 min) to cells, the SMA solution was collected and SMALPs isolated from suspension by ultracentrifuge (110,000 *g* for 20 hours at 20 °C) and protein identities determined by mass spectrometry. Collected proteins were digested with trypsin in-solution and subjected to liquid chromatography-mass spectrometry: this identified an average of 73.0 ± 17.4 unique proteins per sample, in three separate experiments (Suppl. Fig. 1). Panther and GOrilla (Gene Ontology Consortium) were used to determine the most likely cellular location of UniProt-identified^15^ proteins recovered in the SMA biopsy. As we had anticipated, a large number of proteins were obtained from plasma membrane-associated cellular locations, particularly extracellular vesicles and exosomes, however an unexpected addition was that proteins were also obtained from intracellular vesicles and cytoskeletal locations (Fig. 3a,b). To explain these findings, STRING analysis of these data, which identifies likely protein-protein interactions in the protein set and thus allows more detailed understanding of protein sub-cellular location, was applied to the SMALPs from CFs (Fig. 3c) and VSMCs (Fig. 3d). This identified the cytoplasm, plasma membrane and actin-bound proteins as the most common origin of proteins recovered, perhaps suggesting that proteins other than transmembrane proteins are sampled by the SMA biopsy technique due to their interaction with membrane components via the cytoskeleton. Further STRING analysis identified a range of individual proteins known to be of extracellular vesicles and their protein-protein interactions, which were obtained from both CFs and VSMCs (Suppl. Fig. 2). While some of the proteins recovered by SMA biopsy were common across both cell types (including several heat-shock proteins: HSPA8, HSPB1, HSP90AA and HSP90AB), proteins characteristic to CFs (vinculin, VCL, P18206) or VSMCs (Alpha actinin 4, ACTA2, P68032) were identified in the corresponding SMA biopsies, demonstrating that the repertoire of proteins receovered reflected the cell source to some extent (Fig. 3e).

### SMA obtains proteins from tissue, without significant cell death

**Figure 4:**
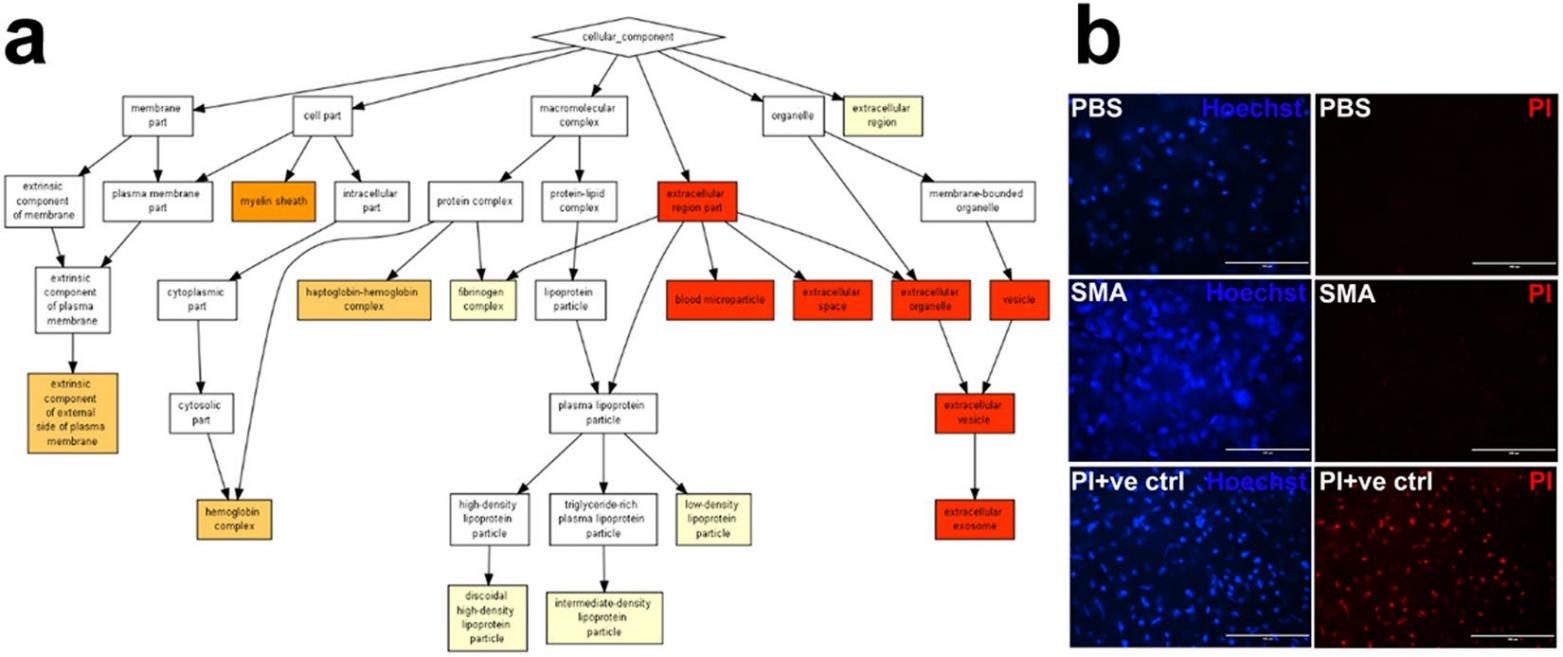
SMA application to intact tissue *ex vivo* was non-toxic and obtained a range of proteins. Mass spectrometry confirmed successful sampling of proteins from *ex vivo* rat aorta following 10 min exposure to 6.25 ppm SMA, with (**a**) Gorilla (GO) mapping of proteins with subcellular locations identifying proteins linked with: extracellular vesicles and exosomes; external plasma membrane; lipoprotein particles. (Colour code for GO map, showing confidence of protein sampling from these locations: *p* > 10^−3^ white; *p* 10^−3^–10^−5^ beige; *p* 10^−5^–10^−7^ pale orange; *p* 10^−7^–10^−9^ dark orange; *p* < 10^−9^ red). (**b**) The non-toxicity of the SMA sampling technique in intact tissue was confirmed by Hoechst (blue) and propidium iodide (red) dual-staining of aorta after 10 min application of 6.25 ppm SMA, compared to vehicle (PBS) or known toxin (PI + ve ctrl) (bar = 100 µm).

The final study objective was to confirm the efficacy of the SMA biopsy method when applied to intact tissue, using vascular tissue *ex vivo*. For this, adult Wistar rat aorta tissue samples were obtained, washed thoroughly with sterile Dulbecco’s PBS and 6.25 ppm SMA applied to the vessel interior for 10 minutes. The SMA biopsy method in *ex vivo* vessel also obtained a range of proteins, as identified by mass spectrometry, which were correlated with a similar range of cellular locations, namely extracellular vesicles and exosomes; external plasma membrane; lipoprotein particles (Panther and GOrilla analysis, Fig. 4a). Further STRING analysis of these proteins indicated the following subcellular localisation: cytoplasm; plasma membrane; actin associated (Suppl. Fig. 3). Tissue images following dual nuclear staining with Hoechst 33342 (staining all nuclei) and propidium iodide (PI, staining dead cell nuclei only) showed cells were PI-negative in tissue after 6.25 ppm SMA biopsy procedure. This confirmed no impact on cell viability in treated tissue (Fig. 4b).

## Discussion

We have determined that the application of SMA at 6.25 ppm (equal to ~1 µM) for 10 minutes sampled proteins of a broad range of sizes without causing significant cell death, thus demonstrating the efficacy of this technique as a cellular ‘biopsy’ tool. We have further demonstrated that SMA can retrieve proteins in different cell types and from intact tissue without significant cell death. The reagent is able to recover proteins from a wide range of sub-cellular locations, not only the plasma membrane but also the cytosol and actin-cytoskeleton, which further extends its capability as a biopsy tool. This therefore provides a novel tool to advance the detection of cell biomarkers, from passive collection to the active retrieval of them from cells or intact tissue.

Using text mining, we determined that for CFs around 31% of identified proteins either contain a transmembrane domain or are directly associated with the plasma membrane, with another 32% of identified proteins associated with the actin filament network. In VSMCs the numbers are similar: 31% of proteins contain transmembrane domains or are otherwise associated with the plasma membrane, whereas 26% of identified proteins are associated with the actin filament network. It is therefore likely that a substantial proportion of these proteins have either been sampled directly from the plasma membrane, or indirectly through association of the actin filament network with the plasma membrane.

Although previous work to achieve sampling of intact proteins from live cells has been carried out, this has to date only been achieved using laser dissection techniques^16^. Whilst elegant, the laser dissection approach is difficult to perform at scale, or to internal tissue structures. The achievement of protein sampling without extensive cell destruction using a stable, widely-applicable chemical tool opens the door to possible *in vivo* or clinical utilisation of intact protein capture. To the best of our knowledge, this is the first demonstration of intact protein sampling while retaining cell viability by such a tool.

Most use of SMA to date, in the context of protein chemistry, has focused on its value for analyses of membrane protein biology or structure: we had therefore expected plasma membrane proteins to be sampled in our study, as was indeed seen. However, the collection of proteins found in both peripheral (extracellular vesicles and exosomes) and more central (cytoskeleton, cytosolic and intracellular organelles) locations indicates that this technique potentially samples from a wider range of cellular areas than had been initially anticipated. This may offer a wider scope for the potential of SMA to sample cell biomarkers of development or dysfunction.

The explanation for the ability of SMA to sample proteins from such a wide range of locations is uncertain, although it is plausible that the obtained nanodiscs would include proteins adherent to their formerly-intracellular surface. This interpretation is supported by the presence in all SMA samples (from both CFs and VSMCs) of moesin (MSN, P26038; linking cytoskeletal structures with the plasma membrane) and of filamin A (FLNA, P21333; anchoring transmembrane proteins to the cytoskeleton).

Beyond this, the foundational data presented here, that this ‘biopsy’ technique can be effectively applied to intact tissue *ex vivo* without tissue disruption or cell viability loss, open the possibility of its use in an *in vivo* setting. The data presented here of tissues ‘biopsied’ by SMA *ex vivo* offer only a limited model of tissue response regarding potential *in vivo* SMA application, due to the substantial differences to the *in vivo* setting. It is however notable that this chemical (in anhydride form, combined with DMSO) has been used for many years as a contraceptive agent^17^, at much higher concentrations than required for protein sampling in this study, with phase I and II human trials completed^18,19^.

While this technique sampled cell-specific proteins from each of the cell types examined, it was also identified that a broad range of proteins were consistently sampled from both cell types. Notably, several proteins which have been identified as possible disease biomarkers (HSPA8^20^, HSPB1^21^, HSP90AA^22^ and HSP90AB^23^) were found to be consistently sampled in both cell types. This suggests that this technique could obtain these potential biomarkers from target cell populations also, for the diagnosis or staging of disease.

The novel biopsy technique opens a new avenue towards the recovery of useful biomarker information from cells and tissues. It is well known that other amphipathic polymers of related structure exist, which also harvest proteins from biological membranes^24,25^. It is entirely possible that these polymers could biopsy in a similar way to SMA, but may recover a different repertoire of proteins from the specimen. This would further extend the potential of the technique demonstrated here.

The ability to exploit this protein sampling technique, which has been demonstrated for the first time here in both cells and tissue, by directing its use towards the detection of pathological markers at a preclinical stage, could be of immense value. The potential to form a personalised evidence-based stratification of individual risk could be utilised for a range of common and severe diseases, with the vascular complications of diabetes mellitus a representative example.

## Methods

### Human tissue sample collection

Primary human cardiovascular cells of two types were examined: cardiac fibroblasts and vascular smooth muscle cells, each isolated from donated samples of human tissue. Ethical approval was obtained from the NHS Health Research Authority: Yorkshire and the Humber - Leeds West Research Ethics Committee (reference CA01/040), with all procedures carried out in accordance with local and national guidelines and regulations. Informed donor consent was obtained for the use of each human tissue sample.

### Cardiac fibroblast isolation

The CFs were isolated from myocardial tissue samples, obtained during the routine progress of cardiac bypass surgery in adult patients. Samples were digested and the cells of interest isolated according to established methods^13^, prior to being maintained and examined *in vitro* as adherent cells.

In brief, myocardial tissue underwent mincing, followed by digestion. Digestion of tissue was achieved by incubation with 800 U/ml collagenase type II solution (Worthington Biochemical Corporation, Lakewood, NJ, USA) in Dulbecco’s modified Eagle medium (DMEM) containing 0.05% bovine serum albumin (BSA) for 4 hours at 37 °C. Cells were pelleted by centrifugation, washed with DMEM/BSA, and re-suspended in full growth medium. Cells were then plated onto cell culture flasks in a humidified incubator at 37 °C, 5% CO_2_ for 30 minutes, allowing CFs to adhere to culture vessels. After this, all non-adherent cells were removed and experiments performed on early passage cells.

### Vascular smooth muscle cell isolation

Tissue samples were long saphenous vein pieces, obtained during the routine progress of cardiac bypass surgery in adult patients. Samples were then dissected and the cells of interest isolated according to established methods^14^, prior to being maintained *in vitro* as adherent cells.

In brief, VSMCs were obtained by producing vein tissue explants approximately 1 mm^3^ in size, then maintained in medium (as described below) in a humidified incubator at 37 °C, 5% CO_2_ for 1–2 weeks. After this time, VSMCs were seen to be migrating from explants. Explants were then maintained for another 2–3 weeks and cells passaged with remaining explant tissue discarded. Cell phenotype was confirmed by immunostaining.

### Cell culture

Both CFs and VSMCs were plated and maintained in the following medium: Dulbecco’s Modified Eagles’ Medium (DMEM, cat. no. 21969); 10% Foetal Calf Serum (FCS); 1% Glutamax; 1% Penicillin-Streptomycin; 0.1% Fungizone (all Life Technologies; percentages v/v). Plating densities and maintenance conditions were consistent for both cell types. Cells were plated at densities of: 20,000 cells/well in 24-well plates for viability assays, or 80,000 cells/well in 6-well plates for protein sampling. Cell were maintained prior to experiments in a humidified incubator at 37 °C, 5% CO_2_.

### Rat tissue isolation

For samples of intact vascular tissue, 200 g male Wistar rat aortas were freshly obtained after sacrifice (animals sacrificed by Home Office Schedule 1-approved methods). Tissue was dissected out from the proximal arch to the aortic bifurcation and washed thoroughly in sterile Dulbecco’s PBS. All sacrifice methods were carried out in accordance with Home Office guidelines and regulations for procedures involving protected species. No protocols were carried out on animals before sacrifice.

### Tissue culture

For cell viability studies, rat aorta was cut into 1 cm lengths after washing and cut along the length to expose the interior lumen surface of the vessel. Explanted rat aorta tissue samples (1 cm length) were maintained in medium as for cultured human cells, but with supplement of medium with 30% FCS^26^. Tissue segments were maintained in 24-well plates prior to experiments in a humidified incubator (37 °C, 5% CO_2_) prior to use in assays.

### Styrene maleic acid application

Stock SMA solution, 2.3:1 ratio, 25% concentration (w/v) (SL30010), was obtained from Polyscope polymers B.V. (Geleen, the Netherlands). This was applied to cells at final concentrations ranging from 1.25 to 25 ppm, diluted in Dulbecco’s PBS containing Ca^2+^ and Mg^2+^ (Life Technologies) immediately before application. These concentrations were applied to both human cell culture treatments and in rat aorta tissue viability studies. SMA applications were performed for 10 minutes in a Binder humidified tissue culture incubator (37 °C, 5% CO_2_). To provide a positive control for cell membrane lysis, 0.01% Triton-X 100 in Dulbecco’s PBS (v/v) was applied to cells or tissue segments (for 10 minutes under the same conditions as for SMA application). For full length dissected rat aorta, tissue samples were rinsed and maintained in tissue culture grade Dulbecco’s PBS, followed by 10 minutes recycled 5 ml solution ‘flush’ biopsy with SMA or Dulbecco’s PBS for SMA biopsy and control respectively, and staurosporine for cytotoxicity-positive control.

The SMALP nanodisc suspensions containing sampled proteins were aspirated and placed on ice for immediate use, or were stored at −80 °C for later analysis.

### Assays of membrane integrity and cell viability

Medium was replaced with 10 µM calcein-AM (GeneCopoeia) in DMEM for 30 minutes, in a humidified incubator (37 °C, 5% CO_2_), before medium was aspirated and cells washed with Dulbecco’s PBS to remove all extracellular calcein-AM and medium. Cells were treated with a range of SMA concentrations as described, then returned to normal culture medium. For analysis, cells were placed in a fluorometric scanner (Varioskan Flash; excitation 495 nm, emission 515–525 nm).

For MTT assay of cell viability, cells were returned to normal culture medium after SMA treatment and maintained in a humidified incubator for 24 or 72 hours (37 °C, 5% CO_2_). After this, 0.5 mg/ml MTT (Sigma) in culture medium was applied for 60 minutes (37 °C, 5% CO_2_), then the cells lysed by applying solubilising solution (4% 1 M HCl in propan-2-ol/isopropyl alcohol, 300 µl/well) and pipetted thoroughly: this lysed cells and distributed the crystals formed by the MTT reagent as previously described^27^. The plate was then transferred to a Varioskan Flash plate reader and absorbance recorded at 570 nm (with reference absorbance 630 nm).

### SDS-PAGE

The optimal SMA concentration for cell protein sampling (6.25 ppm) was used to collect proteins within SMALP nanodiscs from biotinylated cells, using a cell-impermeable biotinylation reagent. Cells were biotinylated with 0.2 mg/ml biotin-XX SSE/DMSO (Life Technologies) in Dulbecco’s PBS (without Ca^2+^ and Mg^2+^) at room temperature. Protein-SMA samples were mixed with sample loading buffer (0.23 M Tris–HCl pH 6.8, 24% v/v glycerol, 120 μM bromophenol blue, 0.4 M DTT, 0.23 M SDS), containing protease inhibitors and 2-mercaptoethanol. This mixture was incubated for 30 minutes at 37 °C then loaded onto SDS-PAGE gels. Proteins were run through 4% stacking gel at 50 V for 20 min, then through 10% resolving gel at 100 V for 2 hours. Biotinylated proteins were identified by Western blotting: by transferring to membranes with methanol, blocking with 5% BSA (Sigma, UK) and incubating with streptavidin-poly HRP (Sigma) in 5% BSA solution. This was followed by electrochemical luminescent (Thermo-Fisher) detection of HRP. Non-biotinylated protein bands were visualised either using protein staining of gels with InstantBlue solution (Expedeon), or used for in-solution mass spectrometry analysis.

In-gel protein staining of samples was quantified using AIDA Image Analyser (Raytest), with these data analysed to confirm equal variances (Brown-Forsythe test) and normal distribution (Shapiro-Wilk test) as part of ANOVA analysis.

### Mass spectrometry

Collected SMA samples were centrifuged at 110,000 *g* for 20 hours at 20 °C in a benchtop ultracentrifuge (Beckman Coulter), to concentrate six-fold. Samples were reduced (10 mM DTT, 1 hour at 57 °C), alkylated (55 mM iodoacetamide, 30 min in the dark) and digested with trypsin (protease to substrate ratio of 1:50, 18 hours at 37 °C). Digested samples were then analysed using an ACQUITY M-Class Ultra-high Pressure Liquid Chromatographer coupled with a Xevo QToF G2-XS mass spectrometer (Waters UK, Manchester). LC separation of the peptide mixtures was performed on an ACQUITY M-Class UPLC (Waters UK, Manchester). 1 µl of each sample was loaded onto a Symmetry C18 trap column (180 µM i.d. * 20 mm) and washed with 1% acetonitrile/0.1% formic acid for 5 min at 5 µl min^−1^. After valve switching, the peptides were then separated on a HSS T3 C18, 75 µm i.d. × 150 mm analytical column (Waters UK, Manchester) by gradient elution of 1–60% solvent B in A over 30 min. at 0.3 µl min^−1^. Solvent A was 0.1% formic acid in water, solvent B was 0.1% formic acid in acetonitrile. The column eluant was directly interfaced to a quadrupole orthogonal time of flight mass spectrometer (Xevo QTOF G2-XS, Waters UK, Manchester) via a Z-spray nanoflow electrospray source. The MS was operated in positive TOF mode using a capillary voltage of 3.0 kV, cone voltage of 40 V, source offset of 80 V. The source temperature was 80 °C. Mass calibration was performed using [Glu]-fibrinopeptide (GFP) at a concentration of 250 fmol µl^−1^. GFP was also used as a lock mass calibrant with a one second lock spray scan taken every 30 s during acquisition. Ten scans were averaged to determine the lock mass correction factor. Data acquisition was using data dependent analysis with a 0.2 s scan MS over m/z 350–2000 being followed by five 0.5 s MS/MS (m/z 50–2000) taken of the four most intense ions in the MS spectrum. CE applied was dependent upon charge state and mass of the ion selected. Dynamic exclusion of 60 s was used. Data processing was performed using the MassLynx v4.1 suite of software supplied with the mass spectrometer. Peptide MS/MS data were processed with PEAKS Studio (Bioinformatic Solutions Inc., Waterloo, Ontario, Canada) and searched against the Uniprot database (release 2018_02) with entries confined to reviewed records of the appropriate genus. Carbamiodomethylation was selected as a fixed modification, variable modifications were set for oxidation of methionine and deamidation of glutamine and asparagine. MS mass tolerance was 20 ppm, and fragment ion mass tolerance was 0.05 Da. The false discovery rate was set to 1%.

Protein identification was carried out using Uniprot and protein localisation with Panther/GOrilla (Multi Knowledge Project, http://cbl-gorilla.cs.technion.ac.il/). Sample proteomes, as obtained from mass spectrometry, were analysed using GOrilla software to identify the enrichment of protein components from particular cellular locations. Swiss-Prot provided information on cell locations of proteins, thus establishing confidence of our sampling in different locations. Swiss-Prot reviewed proteomes: 20,192 human proteins as of June 21, 2017 (reviewed: yes AND organism: “Homo sapiens (Human) [9606]” AND proteome: up000005640) and 8,017 rat proteins as of November 21, 2017 (reviewed: yes AND organism: “Rattus norvegicus (Rat) [10116]” AND proteome: up000002494). Protein-protein interactions and their subcellular localisation were subsequently analysed by STRING^28^.

### Electron microscopy

Suspensions of SMA and sampled proteins were prepared as previously described in the methods. The negative stain procedure is as described previously^29^. Briefly, 3 µl of suspension was applied to a carbon coated grid which had been glow discharged for 30 seconds. Following this, the protein solution was blotted before the addition of 3 µl uranyl acetate solution (2%) which was left for 1 minute before blotting and the addition of another 3 µl uranyl acetate solution (2%). The grid was then blotted and air dried before imaging. Grids were imaged at a nominal magnification of 49,000 using a FEI Tecnai T12 microscope, operating at 120 Kv fitted with a Gatan Ultrascan 4000 CCD camera.

## Supplementary information

Supplementary Information

## Data Availability

The datasets generated during and analysed during the current study are available from the corresponding author on reasonable request.
