# Supplementary material for: Styrene maleic acid recovers proteins from mammalian cells and tissues while avoiding significant cell death
Source: Sci Rep. 2019 Nov 25;9:16408. doi: 10.1038/s41598-019-51896-1 (PMC6877624; doi:10.1038/s41598-019-51896-1)
Supplement: Supplementary file 1 — Supplementary Information [file 41598_2019_51896_MOESM1_ESM.pdf]

Styrene maleic acid recovers proteins from mammalian cells and tissues while avoiding significant cell death

Andrew J. Smith, Kathleen E. Wright, Stephen P. Muench, Sophie Schumann, Adrian Whitehouse, Karen E. Porter, John Colyer

Supplementary Figures

HUMAN CF-1	HUMAN CF-2	HUMAN CF-3	HUMAN VSMC-1	HUMAN VSMC-2	HUMAN VSMC-3	PBS-1	PBS-2	PBS-3
P60709	P60709	P60709	P68032	P68032	P23528		P01308	Q9P225
P27348	P63104	P63261	P60709	P60709	P09382			
P63104	P12814	P63104	P63261	P63261	P04075			
P12814	P02768	Q9BYX7	P62258	P62258	P00338			
O43707	P08758	P12814	P27348	Q9BYX7	P06733			
P08758	P04075	P02768	P63104	P12814	P60174			
P04075	Q05682	O43707	Q9BYX7	P68133	P14618			
Q05682	P23528	P09972	Q562R1	P62736	P07355			
P23528	P04406	P04075	P12814	P63267	P04406			
P04406	P04792	Q05682	P68133	P02768	P63104			
P50395	P11142	P27797	P62736	O43707	P37802			
P04792	P08238	P23528	P63267	P04075	P09211			
P11142	P07195	Q9Y281	P02768	Q05682	P04792			
P08238	P09382	P04406	O43707	P27797	P30041			
P07195	P06733	P54652	P09972	P23528	P12814			
P09382	P13639	P04792	P08758	P04406	P62937			
P06733	P68104	P11142	P04075	P54652	P21333			
P13639	P21333	P08238	Q05682	P04792	Q01995			
P68104	P09211	P07195	P27797	P11142	P08670			
P21333	P07900	P09382	P23528	P08238	P07437			
Q14315	P01308	P06733	Q9Y281	P09382	P09936			
P09211	P14618	Q5VTE0	P04406	P06733	P07737			
P07900	P00338	P13639	P50395	Q5VTE0	P60709			
P14618	P26038	P68104	P54652	P13639	P00558			
P00338	P35579	P26641	P04792	P68104	P35579			
P26038	P18669	P21333	P11142	P21333	P62987			
P35579	P00558	Q14315	P08238	P11021	P07195			
P18669	Q06830	P11021	P07195	P09211	P08758			
P00558	P62937	P09211	P09382	P07900	P13639			
P13797	P07437	P07900	Q14847	P14618	P62258			
Q06830	P21980	P01308	P06733	P00338	P24844			
P62937	P37802	P14618	Q5VTE0	P26038	P24534			
P30041	P09493	P00338	P13639	P35579	P22392			
O43175	P60174	P26038	P68104	P0CG39	O43707			
P50454	P09936	P35579	P26641	Q6S8J3	P07900			
P41250	P08670	P0CG39	P10768	P0CG38	P63241			
P07437	P07737	Q6S8J3	P21333	P62937	P08238			
P55072	Q01995	Q06830	Q14315	A5A3E0	Q99497			
P21980	P18206	P0CG38	P11021	P00441	P11142			
P37802	P18085	P62937	P09211	Q9BQE3	P29692			
Q9Y490	P07355	A5A3E0	P60842	Q71U36	P18669			
P67936	P22392	P00441	P07900	P68363	P21980			
P60174	P42224	P07437	Q14240	Q6PEY2	Q05682			
P22314	P04264	Q9BQE3	P01308	Q9H853	P04083			
P09936	P13645	P55072	P14618	P68366	Q01518			
O60701		Q71U36	P00338	Q9NY65	Q9BUF5			
P08670		P68363	P26038	Q13748	P55072			
P29692		Q6PEY2	P35579	P21980	P61158			
P30101		P68366	Q8N0Y7	Q9Y490	Q9ULV4			
P13489		Q9NY65	P18669	P07951	Q06830			

O75083		Q13748	P0CG39	P67936	Q14315			
P31946		P21980	P00558	P09493	P68104			
Q16555		Q9Y490	Q6S8J3	P60174	O00299			
P07737		P60174	P13797	P09936	Q9Y490			
Q01995		P09936	Q06830	O60701	P18206			
P18206		P08670	P0CG38	P08670	P26038			
P53396		Q05639	P62937	P07858	Q9Y696			
P18085		P29692	P30041	Q14568	P27348			
P04083		P31946	A5A3E0	Q05639	P35580			
P07355		Q04917	P31949	P29692	P42224			
P61158		P61981	O43175	Q58FF8	P02768			
Q01518		Q16555	P50454	Q58FF7	P18085			
Q00610		P30613	P00441	P07237	P22314			
Q9Y696		P15311	P41250	Q15366	Q16555			
O00299		F8WCM5	P07437	P57721	P13797			
Q9ULV4		P14174	Q9BQE3	P30101	P48643			
P06396		P07737	P55072	P13489	O75083			
P63241		P17812	Q71U36	P68371	P04632			
P46940		P35241	P68363	O75083	P26641			
P22392		P31948	Q6PEY2		P07951			
Q99497		P50990	Q9H853		P50395			
Q13813		Q01995	P68366		P78371			
P42224		P18206	Q9NY65		P53396			
P48643		Q15942	Q13748		P0DMV8			
P40227			P21980		Q16851			
P01023			P37802		Q15942			
P30153			Q9Y490		P07237			
Q04828			P07951					
P59998			P67936					
O15144			P09493					
P17655			P60174					
Q86VP6			P22314					
Q15417			P09936					
Q9Y678			O60701					
P02511			P08670					
P61978								
Q9NZN4								
Q14192								
Q16658								
O14558								
Q14974								
O00410								
P46821								
P29966								
O00160								
Q14764								
P40261								
P68402								
P11216								
P62826								
Q15019								
Q9Y617								
P26639								
P23381								
Q16881								

Supplementary Figure 1. List of proteins sampled by SMA in human CFs and VSMCs, with few or no proteins sampled in the absence of SMA. Mass spectrometry identified 45-105 individual proteins in each sample obtained with 6.25 ppm SMA (applied for 10 minutes at 37˚C in a humidified incubator), based on at least 2 unique peptide identifications for every protein. Proteins per sample: 73.0±17.4 (CF samples); 78.7±3.3 (VSMC samples); 75.8±8.0 (all cell samples).

a

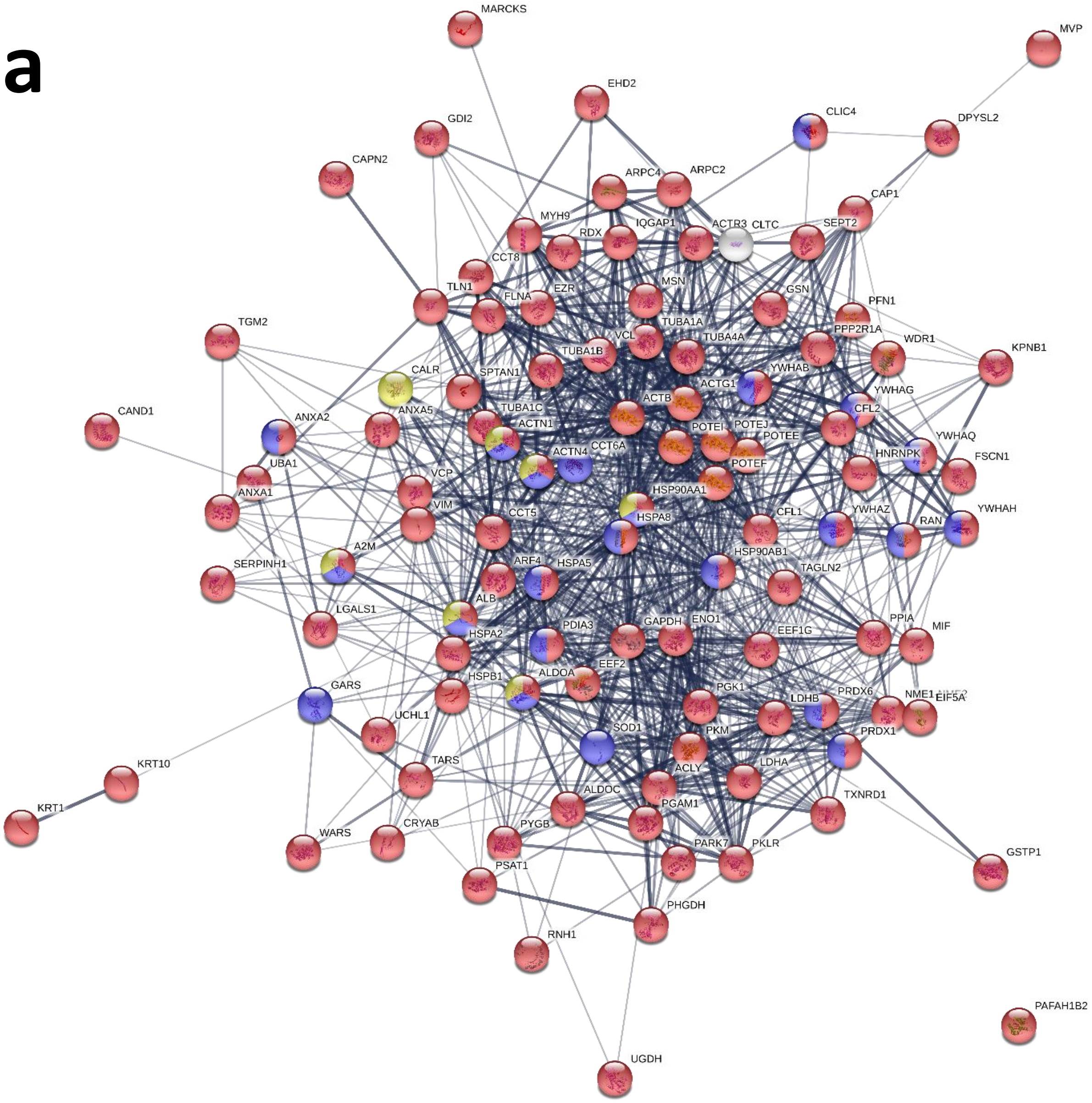

MARCKS
MVP
EHD2
GDI2
CLIC4
DPYSL2
CAPN2
ARPC4
ARPC2
CAP1
MYH9
IQGAP1
ACTR3
CLTC
SEPT2
RDX
CCT8
TLN1
MSN
GSN
FLNA
EZR
PFN1
TGM2
TUBA1A
PPP2R1A
VCL
TUBA4A
WDR1
KPNB1
TUBA1B
CALR
SPTAN1
YWHAB
ACTG1
YWHAG
CAND1
ACTB
CFL2
ANXA2
ANXA5
TUBA1C
ACTN1
POTEI
POTEJ
POTEE
YWHAQ
UBA1
ACTN4
CCT6A
HNRNPK
FSCN1
POTEF
ANXA1
VCP
HSP90AA1
VIM
HSPA8
CCT5
CFL1
YWHAZ
RAN
YWHAH
A2M
HSP90AB1
SERPINH1
ARF4
HSPA5
TAGLN2
ALB
LGALS1
GAPDH
ENO1
PPIA
MIF
HSPA2
PDIA3
EEF1G
EEF2
HSPB1
ALDOA
GARS
PGK1
LDHB
PRDX6
NME1
EIF5A
UCHL1
SOD1
PKM
PRDX1
KRT10
TARS
ACLY
LDHA
ALDOC
KRT1
TXNRD1
PGAM1
CRYAB
WARS
PYGB
PARK7
PKLR
PSAT1
GSTP1
PHGDH
RNH1
PAFAH1B2
UGDH

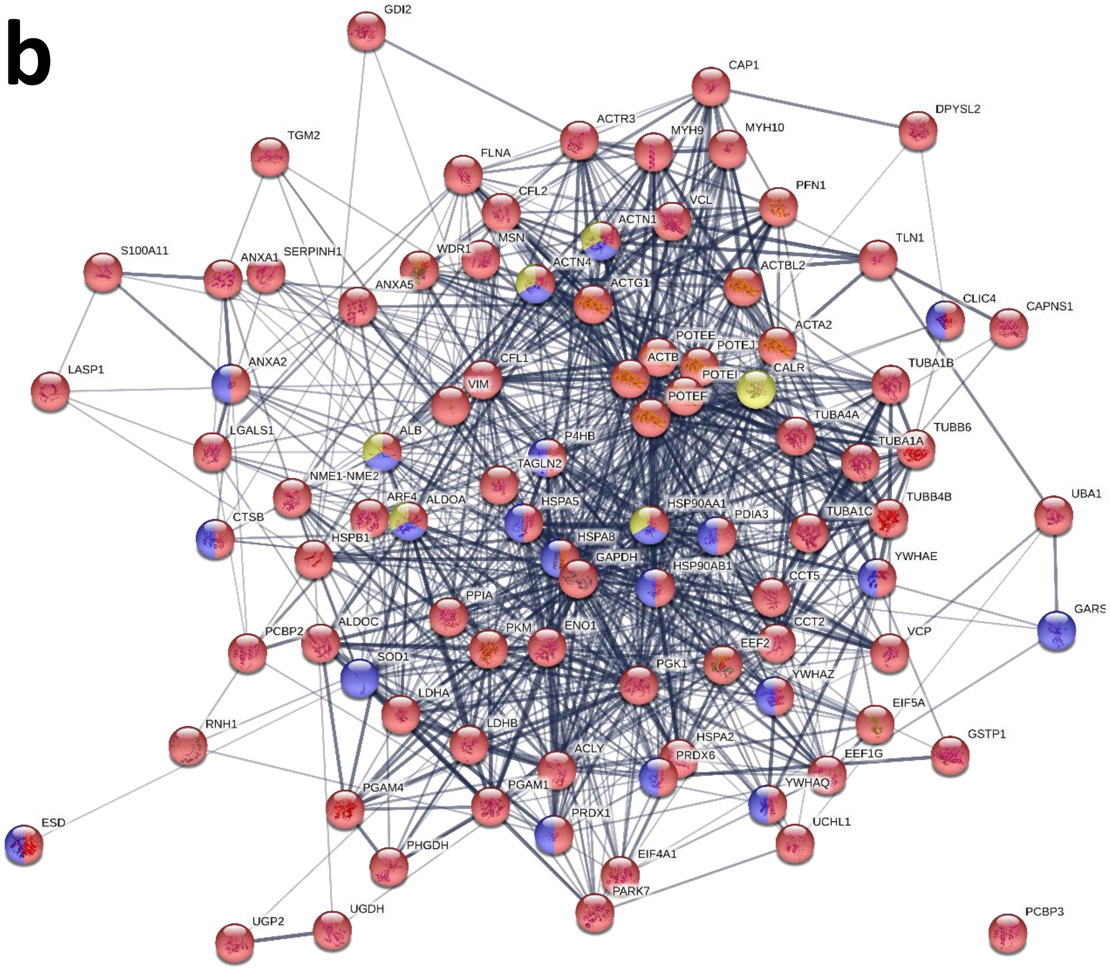

Supplementary Figure 2. STRING analysis of protein-protein interactions of extracellular vesicle-associated proteins sampled by SMA in human (a) CFs and (b) VSMCs. Mass spectrometry identified 110 proteins associated with extracellular vesicles in human CF and 92 proteins in VSMCs that were obtained by 6.25 ppm SMA: membrane bound vesicle (red); cytoplasmic membrane-bound vesicle (blue); cytoplasmic membrane-bound vesicle lumen (yellow). Line thickness indicates confidence of interaction.

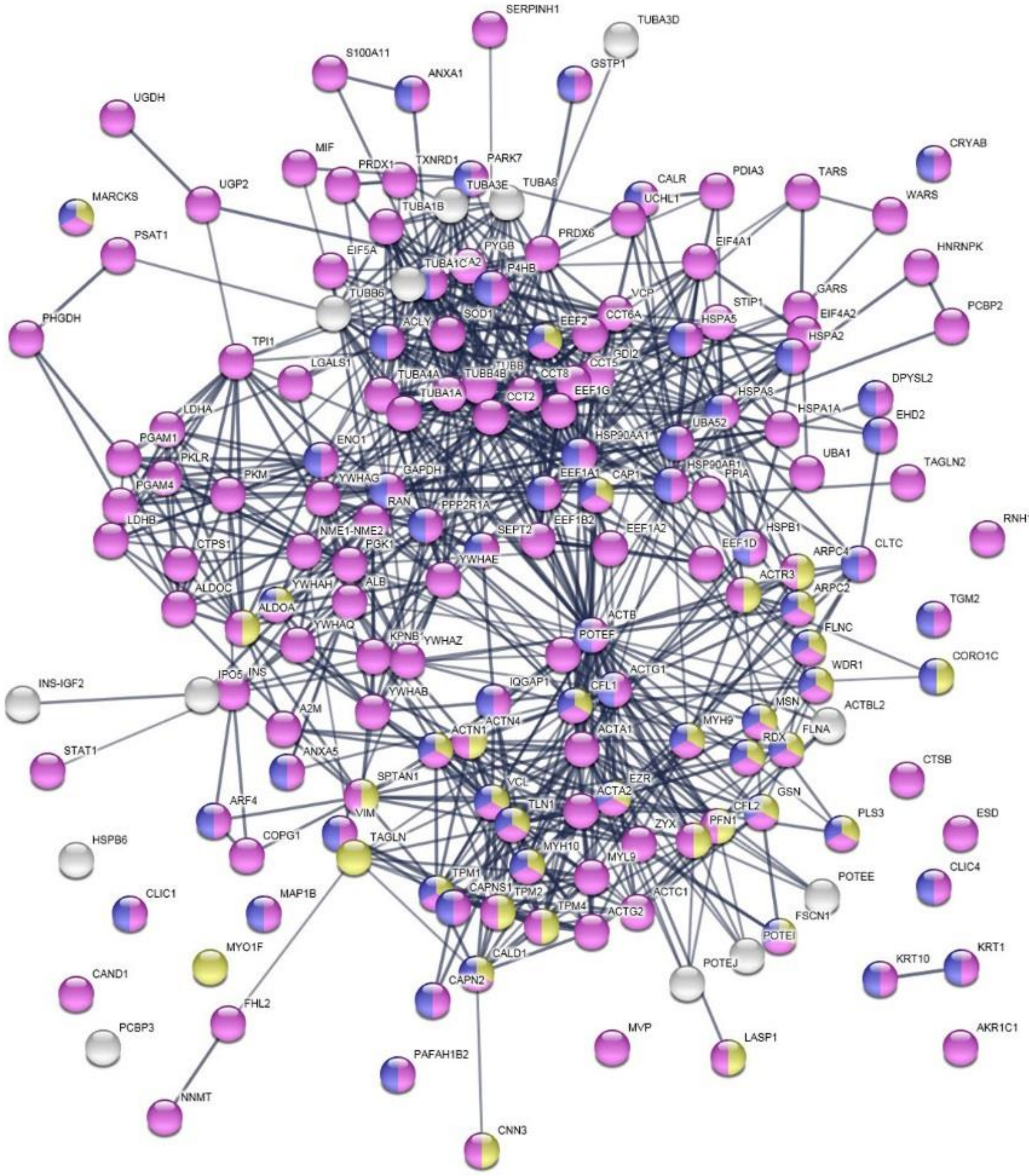

Supplementary Figure 3. Map of proteins sampled by SMA in rat aorta *ex vivo*.

Mapping of identified proteins from intact tissue using SMA extraction by STRING analysis. Subcellular localisation indicated as follows: cytoplasm (purple, 152 proteins); plasma membrane (blue, 61 proteins); actin-associated (yellow, 36 proteins). Line thickness indicates confidence of protein-protein interaction, a confidence threshold of 0.7 (high confidence) was used for all indicated interactions.
